# Infestation mechanisms of two woodborer species in the mangrove *Sonneratia alba* J. Smith in Kenya and co-occurring endophytic fungi

**DOI:** 10.1371/journal.pone.0221285

**Published:** 2019-10-04

**Authors:** Elisha Mrabu Jenoh, Etienne P. de Villiers, Santie M. de Villiers, Sheila Okoth, Joyce Jefwa, Esther Kioko, Davies Kaimenyi, Marijke Hendrickx, Farid Dahdouh-Guebas, Nico Koedam

**Affiliations:** 1 Kenya Marine and Fisheries Research Institute (KMFRI), Mombasa, Kenya; 2 Laboratory of Systems Ecology and Resource Management, Département de Biologie des Organismes, Université Libre de Bruxelles (ULB), Brussels, Belgium; 3 Laboratory of Plant Biology and Nature Management (APNA), Ecology & Biodiversity, Vrije Universiteit Brussel, Brussels, Belgium; 4 KEMRI-Wellcome Trust Research Programme, Kilifi Kenya; 5 Pwani University Department of Biochemistry and Biotechnology, Kilifi Kenya; 6 University of Nairobi (UoN), Centre for Biotechnology and Bioinformatics (CEBIB), Nairobi, Kenya; 7 National Museums of Kenya (NMK), Nairobi, Kenya; 8 BCCM/IHEM: Scientific Institute of Public Health, Mycology and Aerobiology Section, Brussels, Belgium. Rue Juliette Wytsmanstraat, Brussels, Belgium; US Department of Agriculture, UNITED STATES

## Abstract

Insect damage on trees can severely affect the quality of timber, reduce the fecundity of the host and render it susceptible to fungal infestation and disease. Such pathology weakens or eventually kills the host. Infestation by two insect woodborer species (a moth and a beetle) is causing mortality of *Sonneratia alba*, a wide-ranging pioneer mangrove species of the Indo-Pacific. Establishing the infestation mechanism of the two insect woodborer species is an initial and essential step towards understanding their ecological role in the mangroves and in determining sustainable management priorities and options. Our main objectives were to investigate the infestation mechanism employed by the two insect woodborers which infest *S*. *alba* trees, to establish the occurrence of secondary infestation by endophytic fungi in the infested *S*. *alba* branches, and to explore a control management option to the woodborer infestation. We conducted an external inspection of infested branches in two large embayments in Kenya, Gazi Bay and Mida Creek, and by splitting infested branches we determined the respective internal infestation mechanisms. Infested wood samples from Gazi Bay and Mida Creek were incubated at 28±1°C for 3–5 days to establish the presence of fungi. A survey was conducted in both Gazi Bay and Mida Creek to ascertain the presence of ants on *S*. *alba*. The infestation characteristics of the two insect woodborer species were different. It took 6–8 months for the beetle to kill a branch of 150 cm—200 cm long. For the moth to kill a branch, it depended upon several factors including the contribution by multiple species, other than the moth infestation alone. A total of 15 endophytic fungal species were identified. Two ant species *Oecophylla longipoda* and a Pheidole sp. inhabited 62% and 69% respectively of sampled *S*. *alba* trees in Gazi Bay whereas only Pheidole sp. inhabited 17% of the sampled *S*. *alba* trees in Mida Creek. In summary, we have documented the time it takes each woodborer species to kill a branch, the infestation mechanism of the two insect woodborers, and we hypothesized on the role of two ant species. The presence of several different fungal species was ascertained, and we discussed their possible role in the infested wood. Our results cannot unambiguously associate the woodborers and identified fungi. We recommend further studies to investigate the presence or absence, and if present, the nature of fungi in the gut of the woodborers.

## Introduction

Insect woodborers comprise some of the most serious pests of forest trees world-wide. They inhabit and feed on the inside of bark and/or on the wood [1–8]. Insect woodborers damage trees by tunnelling through the inner bark, cambium and conductive tissue; if the stem is completely girdled, the tree dies at or above the damaged site. Partial girdling reduces tree growth and vigour above the site of attack. Tunnelling and disruption of transport of photosynthates by the canopy weaken the tree, causing leaves and branches to fall [4]. Insect damage can severely affect the quality of timber, reduce propagules produced by the host, as resources are reallocated to compensate for herbivore damage [5–6]. Trees become susceptible to disease and fungal infestation, which can accelerate the weakening and eventual killing of the host [9]. Fungal infestations facilitate woodborer feeding activities The role of Fungi is thought to assist in pre-degrading wood thus enabling a variety of woodborers to feed on the wood components [10,11,12]. In some cases, woodborers ingest live fungal tissue or wood substrate into which fungal enzymes have been secreted so that the enzyme can contribute to the digestion of structural polysaccharides, including cellulose [13]. The insect gut systems do not have the capacity to degrade lignin [14,15] and other aromatic polymers that protect the plants from most forms of microbial attack [10,14,15,16,17] hence rendering wood digestion by insect woodborers a challenge. In other instances, woodborers act as vectors carrying the fungi to the next infested tree host but the woodborer derives no benefit from the fungi [10,11]. In a study on fire-destroyed conifer forest, it was realized that insect infestation acts together with other factors like initial tree vigour, degree of injury caused, extent of infestation and weather, to dictate the probability of plant death following forest disturbance [9]. In terrestrial forests, studies relating to fungal presence in trees have been widely conducted [9,10,11,18]. In comparison, for mangrove forests, very few studies on the presence and the role of fungi in the mortality of insect woodborer-infested trees or branches exist [19,20].

Infestation by two woodborers, a metarbelid moth (Lepidoptera: Cossoidea) of an as yet undescribed genus and the beetle *Bottegia rubra* (Aurivillius) (Cerambycidae: Lamiinae), is causing mortality of infested *Sonneratia alba* J. Smith, a wide-ranging pioneer mangrove species of the Indo-West Pacific occurring in the intertidal areas mostly at the waterfront position, in diverse mangroves in Kenya [21,22]. Infestation results in the death of infested branches and death of individual trees. It has also contributed to the death of a relatively large area of *S*. *alba* in Gazi (Kenya), mainly where other challenges like sedimentation (increased surface elevation with sand and silt on the *S*. *alba* stand) contributed to the further weakening of the stand [23]. An aerial survey conducted in 2002 along the Kenyan coastline revealed that infestation was spreading north towards the mangroves of Somalia [22]. A follow up survey conducted in 2012 found that infestation affected only *S*. *alba* and that it had spread along the entire Kenyan coastline. However, the two insect woodborers had different spatial niche distributions along the Kenyan coastline. In areas where their ranges overlapped, such as Gazi, Tudor, Ngomeni, and Lamu, the beetle is infesting the lower branches of a tree and the moth the upper branches, above the high tide line[22,24].

This paper reports on the infestation mechanism employed by the two insect woodborer species to infest *S*. *alba* trees and the time taken by the woodborer to kill an infested branch, ant occurrence in the trees, and the occurrence of endophytic fungi in the infested branches. This information can be important since control of an insect pest is largely dependent on the mechanism it employs to infest its host. On the other hand, knowing the presence of co-occurring fungi present in the infested *S*. *alba* branches adds new information and insight to the mechanism of killing or recovery of an infested branch. In addition, we add knowledge on mangrove mycology which is currently understudied in comparison to terrestrial forest mycology [19,20,25,26].

## Materials and methods

### Study sites

Mangrove forests in Kenya cover approximately 54,000 ha, most of which are located in Lamu and Tana River counties [27,28]. According to Kairo et al., [29] there are nine mangrove species in Kenya, predominantly *Rhizophora mucronata* Lamk. and *Ceriops tagal* (Perr.) C.B. Robinson. Mangrove forests in Kenya can display a zonation pattern of the mangroves also found elsewhere in eastern Africa: the seaward side is occupied by a *Sonneratia-Rhizophora*-*Avicennia* (tall) assemblage, followed by *Rhizophora-Bruguiera-Ceriops* in the mid-intertidal zone and an *Avicennia* (dwarf)*-Lumnitzera-Xylocarpus-*complex on the landward side [29,30]. While this zonation is highly variable from creek to creek and even within a creek or lagoon [30,31], *S*. *alba* has a recurrent typical seaward or creekward position.

**Fig 1:**
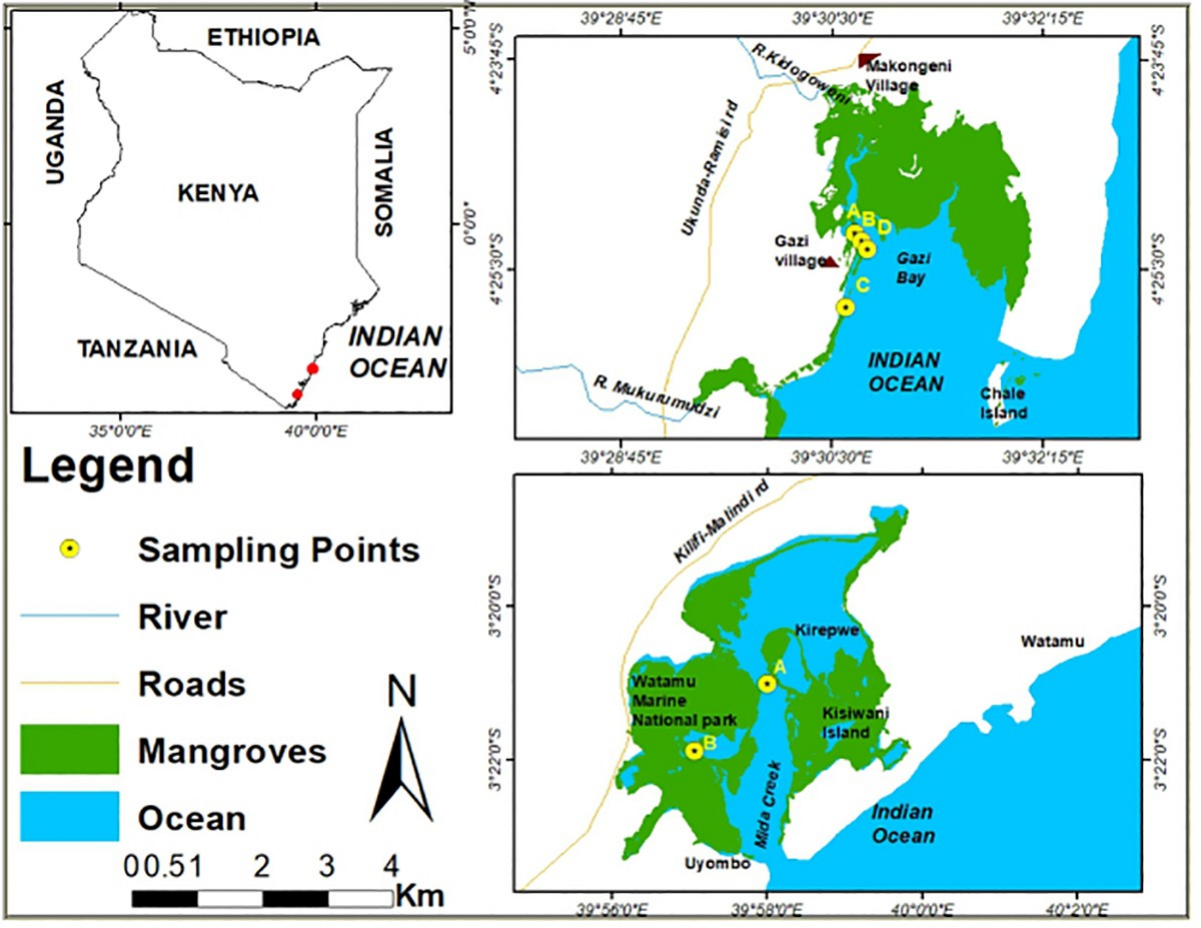
Map of Kenya (inset) with red dots highlighting the Sampling sites (Mida Creek and Gazi Bay). Circles at the sampling sites represents the sampling plots where the study was conducted.

The present work involved (i) a field study conducted along the entire Kenyan coast investigating the infestation mechanism employed by the two insect woodborers; (ii) a field experiment investigating the infestation period needed to kill an infested branch; (iii) an investigation on the presence of secondary infestation by fungi such as to explore their possible ecological role, and (iv) an experiment to test a feasible methods to control beetle infestation. The field experiments (ii,iii and iv) were conducted in Gazi Bay (experiment on the moth woodborer) and Mida Creek (control experiment on the beetle woodborer) along the Kenyan coast (Fig 1). Tree-inhabiting ants were observed in both study areas. Samples for fungal isolation (iii) were taken from both Gazi Bay and Mida Creek to eventually determine the fungal species co-occurring with the woodborers.

### Study site 1- Gazi Bay

**Fig 2:**
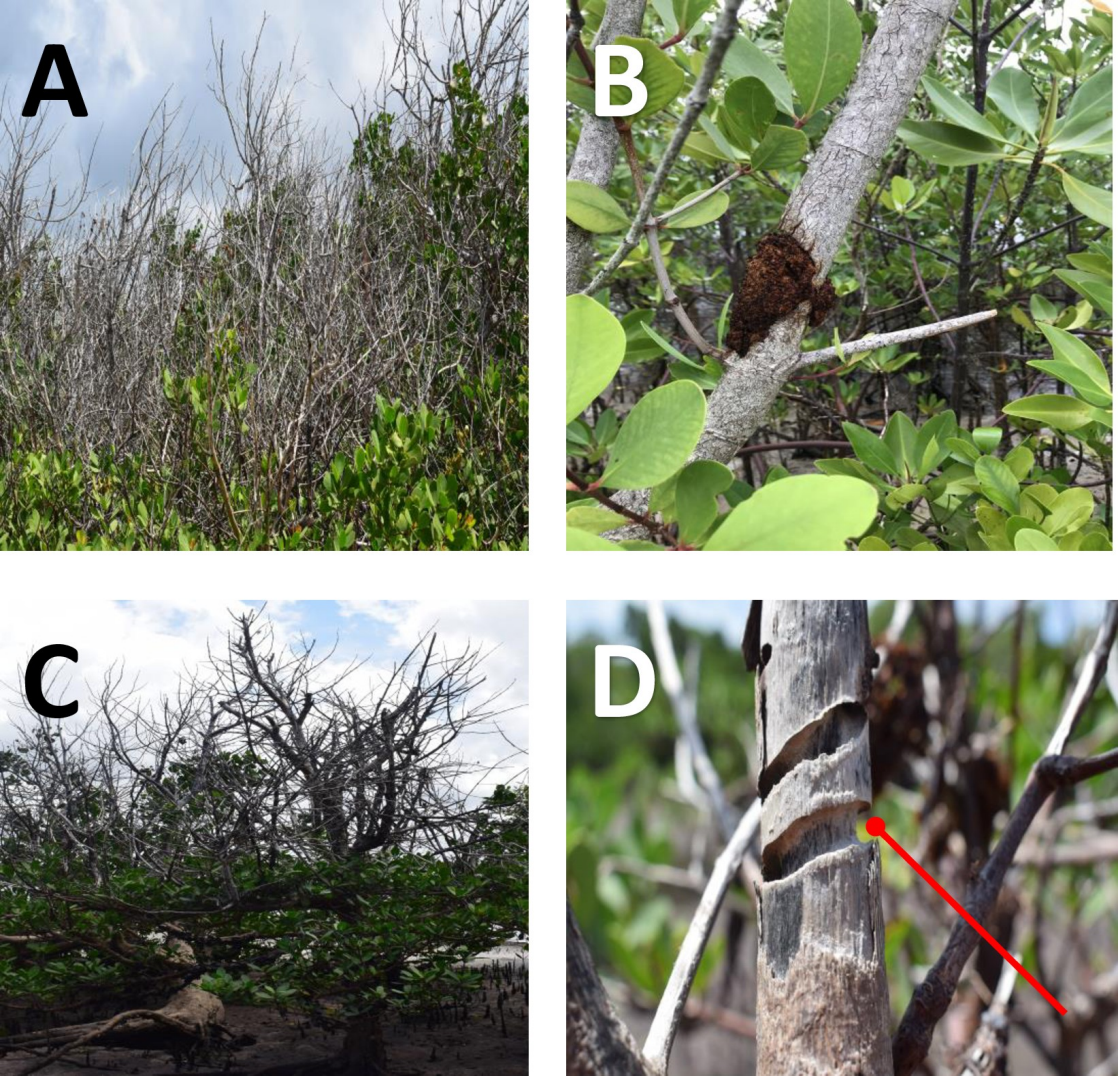
Infested *S*. *alba* forest in Gazi Bay and Mida Creek (C). **(A)** *Sonneratia alba* with dead infested branches in Gazi Bay, **(B)** a picture of a moth woodborer infested branch with frass, **(C)** a beetle-infested *S*. *alba* tree in Mida Creek and **(D)** damage done by *Bottegia rubra* before exiting a *S*. *alba* branch. The arrow shows the base of a branch that has been girdled by the beetle before exiting the infested branch. Apart from infestation by the metarbelid moth, the mangrove of Gazi Bay **(A)** has suffered from sedimentation and infestation by Agelanthus spp. a canopy plant hemiparasite, which is very common.

The field experiment on the moth woodborer was conducted in Gazi Bay (Kenya) located about 55 km south of Mombasa in Kwale County (Fig 1). All mangrove tree species described for Kenya [28,32] are present at Gazi Bay, *Rhizophora mucronata*, *Ceriops tagal* and *Avicennia marina* (Forssk.) Vierh. being most common. Apart from the human-induced degradation of the mangroves in Kenya [33], *Sonneratia alba* in Gazi Bay is experiencing a dieback in some cases due to sedimentation and siltation (Fig 2) [34,35]. However, for two decades *S*. *alba* has been experiencing elevated levels of insect infestation that is contributing to the dieback of infested branches and even killing of some infested trees [21,23,34] within and far beyond the sites with siltation.

Sampling plots A,B,C and D (Table 1) were chosen by considering the availability of a relatively dense *S*. *alba* forest since in the two study sites in particular, and Kenya in general, *S*. *alba* forms only small fringing areas in the mangrove [28,36]. However, it is often dominant at the mangrove’s water edge and on mudflats. Plot A was composed of two sections, one with large old *S*. *alba* trees and the other part with *S*. *alba* forest which was replanted on an originally forested site, 8 and 5 years at the time of observation, respectively. Plot B was mainly composed of old *S*. *alba* trees. In addition to insect infestation, this plot has been affected by sedimentation and hence lost several trees. Plot C was a 15 year old *S*. *alba* plantation. Plot D was composed of a mature, naturally grown *S*. *alba* forest located alongside the main creek in Gazi Bay.

**Table 1:** A table of GPS co-ordinates of the sampling sites and plots used in this study.

Sampling plots/sites	GPS points
**A**	04°25'901''S 039°30'676''E
**B**	04°25'589''S 039°30'789''E
**C**	03°21'100''S 039°58'124''E
**D**	03°21'100''S 039°58'124''E
**Mida Creek (A)**	03°21'S 039°58'E
**Mida Creek (B)**	03°34'S 039°96'E
**Gazi Bay**	04° 44'S 039° 51'E
**Mida Creek**	03°20'S 039°57'E

### Study- site 2-Mida

The field experiment on the beetle was conducted in Mida Creek (Kenya) located 25 km south of Malindi and 100 km north of Mombasa (Fig 1). The study site was chosen for its high incidence of the beetle woodborer infestation and because it is an area where initial studies on this particular insect infestation have been conducted [21] thereby giving the opportunity for validation and continuity. Mida Creek lies in a planigraphic area of 31.6 km^2^ [37] that is under government protection for conservation purposes [36,37].

To investigate the infestation strategy of the beetle in the mangroves of Mida Creek, plots were laid following the same design as in the first detailed study site in Gazi Bay. However, since the *S*. *alba* forest of Mida Creek forms a narrow band, only two specific sampling plots were available for actual sampling *i*.*e*. Plot A and Plot B. Plot A was composed of a narrow band of relatively tall *S*. *alba* mangrove (average height 7.0 m and average diameter D_130_ (*sensu* Brokaw & Thompson, 2000) [38] of 9.74 cm (n = 40) whereas B was composed of relatively young *S*. *alba* (average height 5.5 m and D_130_ 8.48 cm, n = 40).

### Infestation mechanism

To investigate the infestation mechanism employed by the two woodborers, external inspection of infested *S*. *alba* branches was conducted in Vanga, Gazi Bay, Tudor Creek, Mtwapa Creek, Kilifi Creek, Mida Creek, Ngomeni and Kiunga in Lamu county (Fig 1), which is representative of the entire coastline of Kenya. After the external inspection for infestation, samples of infested branches with insect activity and dead branches were randomly collected for further investigation of the infestation mechanism employed by the woodborers. In each of the collected samples the number of larvae in one infestation location, the internal diameter of the entry/exit burrow, the number and the length of the feeding galleries, the length of the branch segment the insect feeds on measured from entry point to the exit burrow, the presence or absence of other developmental stages (e.g. larva, pupa or nymph), and the feeding direction (either upward, horizontal or downward direction in relation to the entry burrow) were determined. For the moth, the diameter of the branch was determined at 5 cm above the insect’s entry point since at this point there was minimal damage to the branch thus giving reliable measurements of the branch. Whereas for the beetle, the diameter of the branch was determined at the central point of the infested branch. This is because the infested branches were not physically damaged on the outside at this point.

### Minimum time taken by a metarbelid moth to kill a branch

In Gazi Bay 20 moth-infested branches from 20 randomly taken *S*. *alba* trees were used to investigate the minimum time taken for the moth to kill a branch. For each of these infested branches, a non-infested branch placed in a similar canopy position on the same tree and of similar size to the infested branch was used as a control. A red tape was used to mark the entry point of the insect. All sub-branches (from the infestation point towards the canopy) arising from the sampled branches (marked point) were counted to track growth of the branch. For a control branch, a mark was put on a branch at a position similar to the position of the infestation point, and all the sub-branches arising from the control branch were also counted. The number of living sub-branches was counted and recorded monthly for eleven months. In case of death of a branch (i.e. completely dry branch with no leaves and presence of entry/exit hole that does not have frass), the cause of death was established as either cutting, insect damage or breakage by other causes.

### Minimum time taken to kill a branch—Mida Creek (beetle *Bottegia rubra*)

In Mida Creek, 30 newly beetle-infested branches from 30 randomly taken *S*. *alba* trees were used to investigate the minimum time taken for the beetle to kill a branch and the average length of a branch the beetle larvae destroy before they emerge as adults. For each infested branch a non-infested branch on the same tree was marked to serve as control. Controls were taken in a similar canopy position and were of similar length and size as much as possible (200 cm long). All sub-branches (from the tip of the branch to the end of the 200 cm length downward) arising from the sampled branches were counted to track growth of the branch. This was done since the beetle infests the branch from the uppermost position of a branch eating the wood down towards the base of the branch. The number of living sub-branches was counted and recorded monthly for eleven months.

### Survey of the occurrence of ants in Gazi Bay and Mida Creek

To investigate the occurrence of ants, a survey was conducted in the two sampling sites. Nine (9) randomly taken S. *alba* trees from each of the four sampling plots (A, B, C and D) in Gazi Bay were surveyed for the occurrence of ant species (ants were considered present on a tree after 5 sightings on the same tree or the ants were ≥20 in one sighting). In Mida Creek, 34 randomly taken *S*. *alba* trees were identified for the survey. In both sites, samples of observed ants were put in an Eppendorf tube with 95% ethanol and transported to the National Museums of Kenya (NMK) for identification using the reference collection in the Entomology Department. Percentage occurrence of the ants in both sites was calculated.

### Fungal isolation from insect-infested mangrove wood—Gazi Bay and Mida Creek

In each of the two sampling sites, six trees were identified for sample collection. From these trees, samples of an infested segment of dead branches (ID) (branches that died from the infestation) and of an infested segment of recovered branches (IR) (infested branches that recovered from infestation) were cut. A segment of a non-infested branch was also cut from all the chosen trees to be used as a control sample. Care was taken to ensure that the control samples resembled the infested samples in terms of canopy position and size of the branch. Each collected sample from Gazi Bay and Mida Creek was then put in a separate plastic zip bag and placed in a cooler box for transportation to the laboratory.

### Fungal isolation

Fungal isolation was conducted at the Kenya Marine and Fisheries Research Institute (KMFRI) laboratory in Mombasa, Kenya and in the University of Nairobi (UoN), Centre for Biotechnology and Bioinformatics (CEBIB, Nairobi). The samples from KMFRI were sent to BCCM/IHEM (Belgium) for species identification using Matrix Assisted Laser Desorption Ionisation–Time of Flight Mass spectrometry (MALDI-TOF MS). DNA was extracted from the isolates obtained at CEBIB (Nairobi) and PCR amplification (ITS-PCR) for genomic identification was done at Pwani University (PU) in Kilifi. DNA sequencing of the PCR products was outsourced to Inqaba Biotec^TM^, South Africa.

In the laboratory, samples were separately washed in distilled water to remove the debris attached to outer surface and to avoid contamination. Samples were surface-sterilized in 70% ethanol by dipping for 10 seconds followed by 3–4 serial rinsing in sterile distilled water (SDW). The samples were then dried in sterile blotting paper under sterile conditions. Aseptically the samples were then chopped into 1 cm sections and each time the saw blade was cleaned using 70% ethanol before using it on another sample. Sections were further chopped aseptically using a sterile chisel into smaller portions to expose the walls of the galleries. The sterility of the chisel was maintained by dipping it in 70% ethanol for 1 minute and then flaming using a burner, then cooled in SDW. Random chopping was used for fungal isolation of the control samples. Using sterile forceps, the chopped pieces were placed carefully on water agar media, incubated at 25°C for three days. The growing hyphae were observed, and single hyphae isolation made using a sterile pin under sterile conditions. The single hyphae were transferred onto Potato Dextrose Agar (PDA) enriched with 1 ml of lactic acid (SR0021) to each 100 ml of the medium, in Petri plates. This was done under aseptic conditions to avoid contamination at different positions allowing space for growth of the fungi. The fungal isolates were incubated at 28±1°C for five days and the culture morphology observed. Changes in the culture morphology were observed under the same growth conditions until 21 days. Purity plating was done after sorting and purifying the different fungal isolates on the plates based on their morphology, colour, size and shape [39]. The different colonies were inoculated in duplicates at temperature of 28±1°C for 5 days onto other Petri-plate containing PDA. Taxonomic identification was conducted using molecular methods (DNA sequencing; see below) and MALDI-TOF MS. There are arrangements in place to deposit samples in the culture collections of the National Museum of Kenya (NMK).

### DNA extraction

DNA was extracted from approximately 100 mg mycelia from each fungal isolate grown on PDA, using a Zymo Research^TM^Quick-DNA Fungal/Bacterial Miniprep Kit, following the manufacturer’s protocol.

### DNA amplification, sequencing and identification

The fungal internal transcribed spacer (ITS) regions of the ribosomal DNA (rDNA) was amplified using two primer pair sets; ITS4, fITS7 [40] and ITS3_KYO2, ITS4_KYO3 [41]. Amplification was performed in 50 μL containing PCR buffer (20 mM KCl, 10 mM (NH_4_)_2_SO_4_, 2 mM MgCl_2_, 20 mM Tris-HCl, pH 8.4), 200 μm of each deoxyribonucleotide triphosphate, 15 pmols of each primer, c. 100 ng template DNA and 2.5 units of Taq polymerase. The thermal cycling programme was: 3 minutes initial denaturation at 94°C, followed by 35 cycles of 30 denaturation at 94°C, 30 s annealing at 52°C, and 1 min extension at 72°C and a final 10 minutes extension at 72°C. A negative control using water instead of template DNA was included in the amplification process. About 4 μl of PCR product from each reaction (including the control) was examined by electrophoresis at 80 V for 30 min in a 1% (w/v) agarose gel in 1 × TAE buffer (0.4 M Tris, 50 mMsodium acetate, 10 mM EDTA, pH 7.8) and visualised under ultraviolet light after staining with GelRed.PCR products were directly sequenced with primer pairs as mentioned above on an ABI 3730-XL DNA sequencer at Inqaba Biotec^TM^. Forward and reverse reads were quality trimmed and assembled in CLC Genomics Work bench V 9.5.3. Sequence-based identifications were made by searching the assembled consensus sequences with BLASTN in the UNITE database V 7.2, a database of fungal nucleotide sequences [42]. A value of 97% ITS identity was used as a DNA barcoding criterion [43].

### MALDI-TOF mass spectrometry

Strains were cultivated on Sabouraud medium supplemented with an antibiotic (chloramphenicol) for 72h at 25°C. A protein extraction with formic acid and acetonitryl was carried out as described previously [44]. One (1)μL of the protein extract was measured in quadruplicate using the microflex LT mass spectrometer (Bruker Daltonics, Bremen, Germany), using the default settings of the manufacturer. The instrument was calibrated by means of a Bacterial Test Standard (Bruker Daltonics). The spectra were analyzed in MALDI Biotyper v3.0 (Bruker Daltonics) using an in house created database as described previously [45].

### Cellulolytic test

To test cellulolytic activity, fungal isolates from the isolation at CEBIB (Nairobi) were grown on PDA supplemented with 0.5% Na-carboxymethyl cellulose (CMC) for three days at 25°C. Plates with fresh mycelial growth were flooded with 0.1% Congo red and destained with 1M NaCl. Plates were left at room temperature for 15 minutes to allow clearing of the medium around the fungal inoculum, an indication of cellulolytic activity.

### Ligninolytic test

Ligninolytic activity was also tested on the fungal isolates isolated at CEBIB (Nairobi). They were grown on malt extract agar and PDA amended with 0.025% and 0.2% guaiacol, respectively. The concentrations were based on reported studies [46] where they worked well for ligninolytic activity screening. The plates were incubated for three days to allow development of a coloured zone around and below the fungal inoculum as a sign of ligninolytic activity.

## Results

### Infestation mechanism

The infestation mechanisms of these two insect woodborer species were different (Table 2). On average, the moth attacked branches situated from a minimum of 159.5 cm (n = 42) height from the ground (above ground tidal height in Gazi Bay is 165 cm) and branches thicker than 1.5 cm (n = 120) in diameter. At the entry/exit point it produced brownish frass that was held together by silk threads produced by the larvae. The exit/entry hole and the external feeding galleries are visible once the frass was removed from the infestation point. The insect also girdled the bark of the branch. Leaves of an infested branch gradually wilted and turned brown then fell off gradually after infestation. Larvae of the moth practiced multiple infestations on both the branch and entry point in a branch, i.e. one branch could be infested in several locations and one entry point could be shared by several larvae. The higher the intensity of infestation on a branch, the higher is the likelihood of its manifestation. A single infestation point or single larvae could undergo the entire life cycle without any visible manifestation on the branch. The larvae either made a single feeding gallery or multiple galleries that were situated anywhere within the wood tissue and the pith of the branch. The galleries in the wood did not follow a particular feeding direction but were rather haphazardly oriented. The length of the feeding galleries for the moth ranged from 1.2 cm to 15.3 cm (n = 120).

**Table 2:** Summary of the infestation manifestation strategies employed by the two insect woodborers to infest *S*. *alba* in Kenya.

Moth (Lepidoptera: Cossoidea	Beetle (Cerambycidae: Lamiinae)
Attacks branches thicker than 1.5 cm (n = 120).	Attacks branches of minimum diameter 0.5 cm to 2.1 cm maximum (n = 120)
Produces brownish frass from its entry point hole.	No frass produced at any location on the branch.
Entry point always visible when frass is removed.	Entry point never visible.
Infested branches undergo very gradual chlorosis and gradual defoliation.	Infested branches undergo rapid leaf chlorosis, wilting, browning and eventual rapid defoliation.
Insect practices multiple infestations at both the branch and at the same entry point in a branch, i.e. a branch is infested either once or at several locations and one entry point is shared by several individuals.	A branch is infested only once. Infestation is only in one location within a branch and there is only one entry point for an infested branch.
Larvae either make a single or multiple feeding galleries.	Larvae make only one feeding gallery.
Feeding galleries are situated anywhere within the wood tissue and the pith of the branch.	Feeding galleries are situated at the pith only.
ssThe galleries in the infested branch do not follow a particular feeding direction but are rather haphazardly oriented.	Galleries always have a top-down orientation (galleries are formed from the leaves moving downwards towards the base of branch).
The length of the feeding galleries range from 1.2–15.3 ± 3 cm (n = 120).	The average length of the feeding galleries is 200 ± 9 cm long (n = 120).
It attacks branches of height 159.5 ± 7.2 cm (n = 42 cm) from the ground on average and branches of 1.5 ± 0.3 cm (n = 120) minimum width.	It attacks branches situated 77 ± 6.5 cm from the ground as the lowest height to be attacked (n = 62) trees.

Values after ± refer to the standard error of the various measurements.

The beetle attacked branches with a diameter from 0.5 cm to 2.1 cm (n = 120). At its entry/exit point it produced no frass. It does not use the same point for entry and exit since it first infests the topmost point of the branch when it is still young but exits at the base of the branch when it turns to an adult. Once the larvae start foraging on the branch, the leaves of an infested branch immediately start wilting. This is followed by abrupt and massive defoliation of the branches distal to the position of the larvae on the branch. The beetle does not practice multiple infestations neither at the branch nor at the entry point. This woodborer makes only a single feeding gallery in the pith of the branch. The feeding galleries always had a top-down orientation covering a distance of 180 cm—200 cm long before the adult exited. The lowest branches the beetle attacked were on average situated 77 cm (n = 62) from the ground (above ground tidal height in Mida Creek is 115 cm). Despite the low height of infestation, seawater could not penetrate the infested branch where the larvae were located since there was never a visible entry point to the branch. This allows the beetle to attack young recruited *S*. *alba* trees in both natural systems and in plantations, thus making it a bigger threat to mangrove conservation and restoration efforts than the moth. Also, the ability of the beetle to attack branches below the high water of spring tide level has offered a natural barrier of the target branches between the two woodborers in places where both species occur together.

### Time taken for branches to die upon woodborer infestation

Out of the 20 moth-infested branches in Gazi Bay, only two branches died of infestation by the moth larvae after six months of the experiment. Five branches appeared to show signs of dying, and then recovered from the infestation after the exit of the moth; among these five, two experienced multiple infestations (many larvae using one entry point). Three branches were destroyed by non-infestation causes (most probably by people) before the experiment was over. Apart from one control branch getting infested, all the other control branches (n = 20) survived.

The beetle took seven to eight months to kill a 150 cm—200 cm branch. Among the 30 trees taken, only three infested branches recovered from the infestation. However, this recovery was due to premature death of the beetle larvae. The leaves of the infested branches with active larvae quickly wilted, dried and abscised fast as the larvae progressed downward. All the control branches that recovered from the infestation grew secondary branches below the cut part of the branch showing no further signs of infestation by the beetle.

### Survey of the occurrence of ants in Gazi Bay and Mida Creek

**Fig 3:**
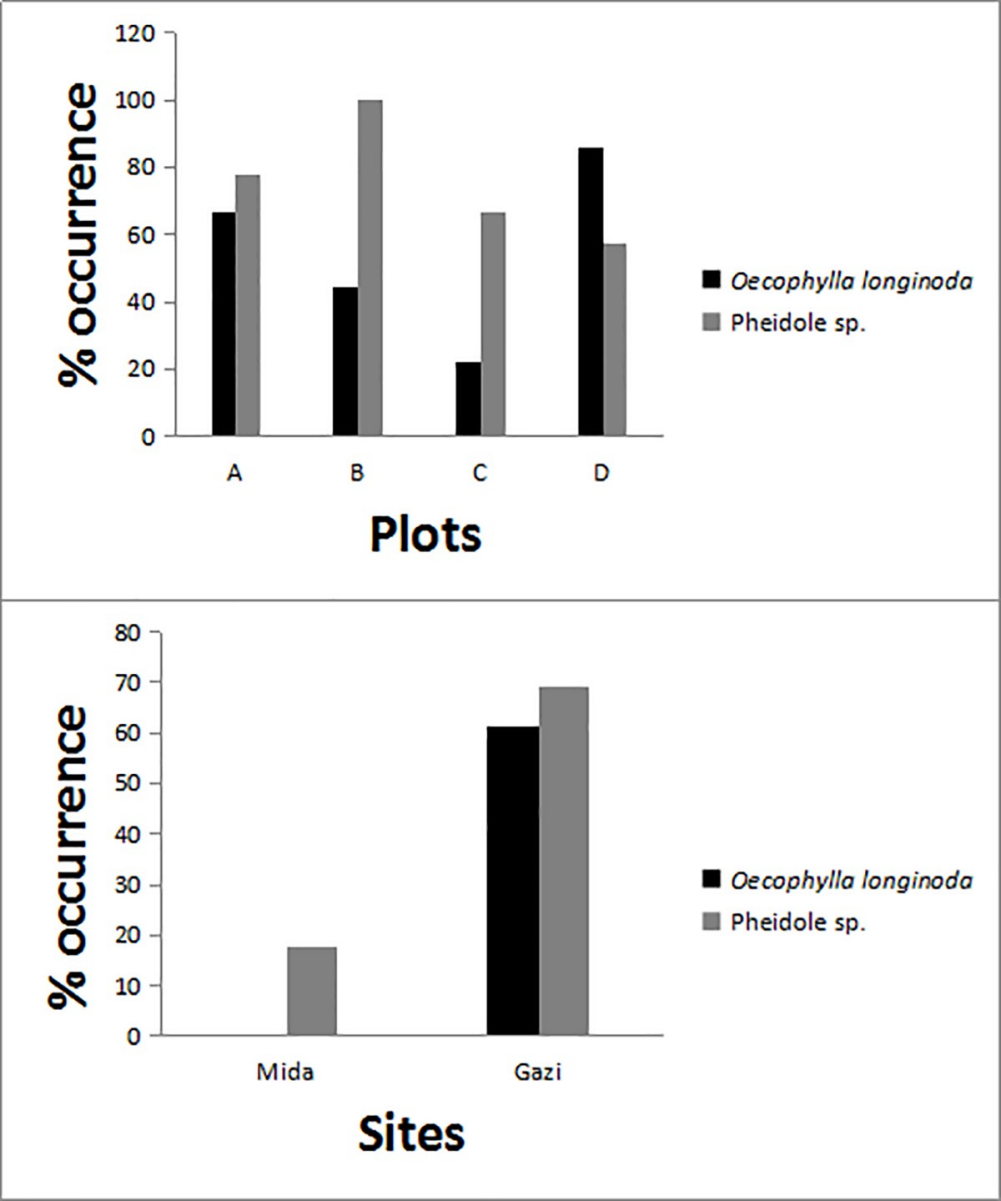
The percentage (%) occurrence of ants (*Oecophylla longinoda* and Pheidole sp.) in the four plots in Gazi Bay (top graph) on the sampled trees. The bottom graph shows the average percentage (%) occurrence of the two ant species found inhabiting *S*. *alba* trees in the two sampling sites (Mida Creek and Gazi Bay). *Oecophylla longinoda* was not recorded in Mida Creek.

Two ant species *Oecophylla longinoda* (Latreille, 1802) and a Pheidole sp. inhabited 62% and 69% respectively of sampled *S*. *alba* trees in Gazi Bay whereas only Pheidole sp. inhabited 17% of the sampled *S*. *alba* trees in Mida Creek (Fig 3).

### Secondary fungal infestation

From the appearance of the purity plated isolates, all the isolates were represented at the two sampling sites and within both the infested dead (ID) branches and infested recovered (IR) branches. In total, 13 fungal isolates were obtained from samples cultured at KMFRI, whereas at CEBIB 17 were obtained. The 13 isolates were sent to Belgium for taxonomic identification by MALDI-TOF MS whereas DNA extracted from the 17 fungal isolates obtained at CEBIB, was ITS-PCR amplified at Pwani University (PU) and sent to South Africa for sequencing.

### Fungal isolate identification using molecular methods

In total, 11 fungal taxa were identified, presented in Table 3. These IT results were the consensus from both forward and reverse reads for all samples except S_1 and S_9, which had only one read each. In addition, the reads from these 2 samples were very short, 70bp and 39bp respectively.

**Table 3:** Summary of the fungal species found in the branches of infested *S*. *alba* trees in Gazi Bay and Mida Creek.

Site	Species	Moth	Beetle	MALDI -TOF MS	DNA Seq.	GBAcc. no.	% sim.	(ID)	(IR)
Gazi	*Aspergillus aculeatus*	✓	-	-	Seq	U65309	99.55	✓	✓
Gazi	*Aspergillus flavus*	✓	-	-	Seq	KP256849	99.66	✓	✓
Gazi	*Aspergillus flavus*	✓	-	-	Seq			✓	-
Gazi	*Aspergillus japonicus*	✓	-	MS	-			✓	-
Gazi	*Aspergillus tubingensis*	-	✓	MS	-			✓	-
Gazi	*Cladosporium cladosporioides	-	✓	-	Seq	MF000937	100	✓	✓
Gazi	*Gibberella intricans*	✓	-	-	Seq	MF099868	99.64	✓	✓
Gazi	*Penicillium sclerotiorum*	✓	✓	MS	Seq			✓	-
Gazi	*Talaromyces australis*	✓	-	MS	-			✓	-
Gazi	*Talaromyces diversus*	✓	-	MS	-			✓	-
Gazi	*Trichoderma inhamatum*	✓	✓	-	Seq	Z68188	100	✓	-
Gazi	*Trichoderma inhamatum*	✓	✓	-	Seq			✓	-
Gazi	*Trichoderma longibrachiatum*	✓	-	-	Seq	MF116270	99.76	✓	✓
Mida	*Aspergillus awamori	✓	✓	-	Seq	LM653116	100	✓	✓
Mida	*Cladosporium asperulatum*	✓	-	-	Seq	LN834357	100	✓	-
Mida	*Penicillium citrinum*	✓	-	MS	Seq	LT558897	99.42	✓	-
Mida	*Penicillium flavigenum*	-	✓	-	Seq	NR:103695	99.66	✓	-
Mida	*Penicillium sclerotiorum*	✓	✓	MS	Seq	KU192990	100	✓	-
Mida	*Penicillium sclerotiorum*	✓	✓	MS	Seq			✓	-
Mida	*Penicillium sclerotiorum*	✓	✓	MS	Seq			✓	-
Mida	*Penicillium sclerotiorum*	✓	✓	MS	Seq			✓	-

* Match obtained from only one short read (S_1 70bp and S_9 39bp) The method of fungal identification and the associated woodborer is shown. Species names of isolated endophytic fungi identified using both molecular methods (DNA sequencing) and MALDI-TOF mass spectrometry (similarity to known DNA sequences and MALDI-TOF mass spectrometry profiles respectively). The symbol (✓) and (-) means presence and absence of that particular entry respectively. ID and IR refer to ‘infested dead’ and ‘infested recovered’, respectively. Where the abbreviations DNA Seq (DNA sequencing) and MS (MALDI-TOF mass spectrometry method) appear, it means the method was used at that particular entry. GB ACC no. stands for GenBank accession number of match. % sim stands for percentage similarity.

A total of 15 different species were identified. Of these, only three, *Penicillium sclerotiorum*, *Trichoderma inhamatum* and *Aspergillus awamori* co-occurred with both the moths from Gazi Bay and the beetles from Mida Creek. Thirteen (13) fungal species (five from the genus Aspergillus, three Trichoderma, two Talaromyces and one species each from genus Penicillium, Cladosporium and Gibberella) co-occurred with the moths, whereas beetles showed co-occurrence of only five different species.

### Cellulolytic test and ligninolytic test

All fungi analysed in this study tested negative for cellulolytic and ligninolytic activity with the techniques used.

## Discussion

### Insect woodborer infestation manifestation on branches of *Sonneratia alba*

**Fig 4:**
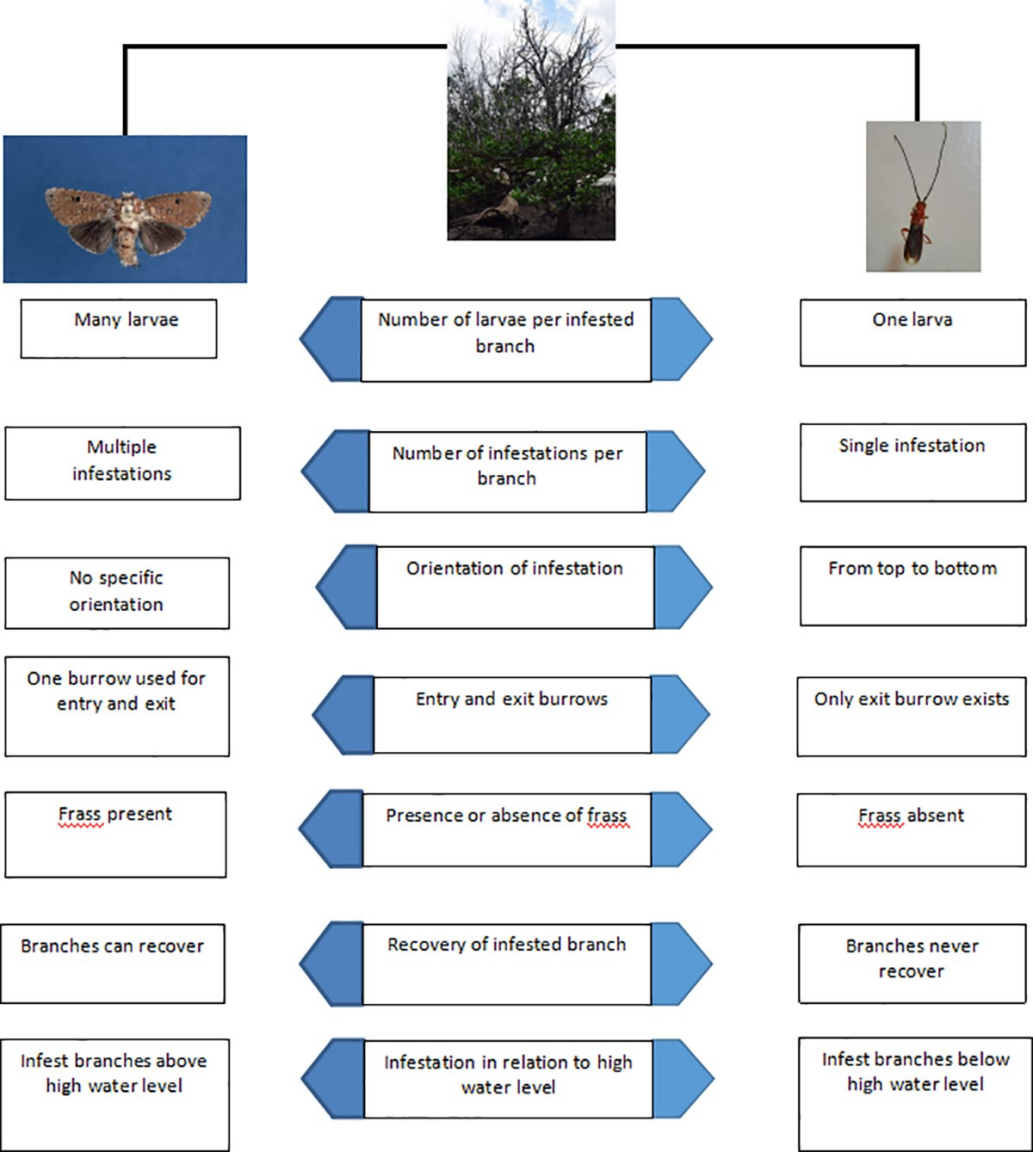
A scheme summarizing the differences of the infestation mechanisms employed by the two woodborers to infest *S*. *alba* in Kenya.

The external manifestation of infestation by woodborers on *S*. *alba* branches sheds light on the kind of feeding and consequently the destruction inflicted by the woodborers onto the host plant (Fig 4). For example, differences in the time taken for the leaves of an infested branch to undergo general chlorosis, to turn brown, to wilt and eventually to drop could be due to the woodborer’s feeding mode. With the metarbelid moth, it takes relatively longer periods for all the leaves to turn brown, wilt and eventually drop. This may be because the larvae do not consume the entire transport vessel system but rather girdle only a section of the bark and feed on a localized section of wood tissues beneath the bark, leaving a section of the bark, cambium and some vascular tissue undisturbed. Hence, the branch can still grow, conduct nutrients and water through the non-destroyed vessels and the phloem. More so, we have observed that some infested branches that had been fully defoliated during infestation were able to recover and develop new leaves after the adults exited the branch. This could be a sign of incomplete destruction of the transport tissue including the phloem, and the cambium tissue. This feeding mechanism allows the metarbelid moth to have several larvae simultaneously at different developmental stages in one entry point and to infest different sections of the same branch without killing it. Thus, this mechanism could be an adaptation where the moth preserves the branch for as long as possible maximally benefitting from its host to ensure its own survival success. Since the metarbelid moth infests *S*. *alba* branches without the full manifestation of infestation by the tree, it is possible that the forest is much more weakened than is actually seen when considering the visible infestation only. This scenario, combined with the other challenges to which *S*. *alba* forests in Kenya are subjected, i.e. coast erosion, sedimentation, infestation by a hemiparasite plant (Agelanthus spp.)[23,47] and sea level rise [48], can hamper the species from providing goods and services to the dependent community such as good timber [49] and its ecological role in coastal protection [50,51] in its pioneering and mudflat position.

The coleopteran woodborer, *Bottegia rubra* feeds at the core of the wood of an infested branch from the top of a branch downwards. As a result, since it feeds on all branch tissues, it utilizes the food reserves of the branch and feeds on the conductive tissues also, thus cutting off nutrient and water supply, hence causing rapid wilting, chlorosis and eventual rapid shedding of leaves by the infested branches. Before *B*. *rubra* matures and moves out of its host branch, the larvae ring the bark at the base of the branch and make an exit hole, thus completely cutting off translocation and regrowth ability by destroying the entire cambium and conducting tissue (Fig 2B). Hence the branch dies (Fig 4). *Bottegia rubra* has a very advanced flight ability that enables it to search for other branches to colonize with ease. The killing of the infested branch by the beetle could also explain the incompatibility between the two insect woodborers since the moth prefers either multiple infestation on a branch, without killing it. Killing the branch at the moth’s exiting would be selected against, since young larvae and eggs are present in the same branch it has infested. Such selection against killing would not be as strong for the beetle, since it has no eggs or larvae left at the branch at the time of exit.

### Occurrence of ants in Gazi Bay and Mida Creek and their possible role

Apart from the survey to ascertain occurrence of ants, and the ant observations made during fieldwork, the role of ants has not yet been studied in depth during this study. Their presence was not yet described earlier, but the activities were noted during our research, hence we include some hypothesis on their possible role in the processes leading to death and/or recovery of infested *S*. *alba* trees. Predation by ants on *B*. *rubra* in Mida Creek and the moth in Gazi Bay, could interfere with the impacts of these woodborers on the branches and trees particularly in Gazi Bay.

These ants, *Oecophylla longinoda* and Pheidole sp., have been observed to actively search for the moth larvae and pupae especially where the frass is old or falling apart, as is always the case when an adult moth leaves the branch. In Gazi Bay, *S*. *alba* trees have numerous colonies of these ant species that prey on the larvae and pupal stages of the woodborers. Predation on larvae by these ants protects the host from the full destructive effect of the moth [8, 52], since the moth may be killed before reaching its most destructive stage or during its most destructive period.

The near absence of these ants in Mida Creek could be explained by the low habitus of the *S*. *alba* trees in this area, the position of the tree within the mangrove formation and the impacts of the tidal range. In Mida Creek, relatively tall *S*. *alba* trees have some colonies of Pheidole sp. that can prey on the larvae and pupal stages of the beetle. On the other hand, ants do not have nests on the low trees as they are avoiding submergence by the daily tides. However, ants have been observed to move from their relatively tall nesting trees to the nearby shorter trees that do not have the ant nests via branches that are intertwined. This has allowed ants to protect both the relatively tall trees that they nest on and the adjacent short trees on which they do not have colonies. However due to submergence and the lack of an entry burrow on the beetle-infested branches, the success of Pheidole sp. in controlling the beetle in Mida Creek is minimal. Perhaps the only way Pheidole sp. can control the spread of the beetle in Mida Creek is by either attacking the beetle shortly before it leaves the branch as an adult, or the eggs during oviposition on the branch. A low success rate in predating on the beetle could explain the current range expansion by the beetle towards the south of Kenya. This illustrates that the role of ants in reducing woodborer impact on *S*. *alba* merits further investigation.

### Minimum time taken for branches to die upon woodborer infestation

It seems there are many factors that lead to either death or recovery of a moth-infested *S*. *alba* branch. They include the possibility of a re-infestation success by successive generations, the size of the branch, predation by ants *Oecophylla longinoda* (Latreille, 1802) [53] and a Pheidole sp., the frequency (number of infestations at a given time) and intensity (the number of larvae present at a given time) of the infestation. A branch would die only when the combined effect of these factors is enough to compete with the recovery potential of the tree. Re-infestation or frequent infestation of a branch happening at alternate times may not cause death but only reduce the growth vigour of a branch. On the other hand, a high intensity of infestation can only develop if the larvae persist for a long duration without being preyed upon by any of the two ant species.

The presence of Agelanthus spp. in the trees’ canopy infesting many *S*. *alba* trees is also consuming water and nutrients from the hosts [47], thus contributing to further weakening of host trees and the eventual death or recovery of insect-infested branches [54]. Therefore, for the moth to kill a branch several other factors, other than the moth infestation alone, is expected to interfere.

The killing of 27 out of the 30 experimental branches in Mida Creek emphasizes the vigour of *B*. *rubra* as an effective woodborer. Infestation success of this magnitude may cause serious destruction to a host species depending on the woodborer population density and growth. In a case where the population of adults is increasing, the host species can quickly be overwhelmed and killed. In some cases, the tree stand is weakened, and this is expected to reduce the ability and efficiency of the host to protect itself against other pests and diseases or to provide metabolic energy to overcome impacts of other disturbance such as sedimentation. Since up to now no natural enemy is known to check the population of *B*. *rubra* in the *S*. *alba* forest in Kenya, the capacity of *B*. *rubra* to cause serious damage to the host appears to be higher than that of the moth, especially since it was realized in a coastal survey (Jenoh *et al*., 2016) that *B*. *rubra* is extending its range both northward and southward along the Kenyan coast [22,24]. However, the magnitude of the destruction that *B*. *rubra* could cause to the host and the forest, is buffered by the long generation time and the long time taken by one insect to kill a single branch.

### Secondary fungal infestation

The moth burrows registered almost three times as many fungal species co-occurring with them, as compared to the beetles, 13 versus 5 respectively. This difference in the number of fungal species associated with each type of woodborer could be explained by the habitat and the different infestation mechanisms employed. Once a woodborer exits a branch, other faunal species colonize and utilize the abandoned burrows as shelter, breeding place and even as feeding areas, especially insects that feed on other insects. As the different faunal species colonize these burrows, they may introduce fungal species that they have collected from different sources, thus inoculating them in the abandoned burrows. When conditions are favourable, the fungal species would then establish in the burrows.

Since the moth infests branches that are higher than the maximum tide level [24], many faunal species can colonize burrows abandoned by the moth without interference from the tide, possibly inoculating many different fungal species. Unlike the moth, the beetle infests branches below the high tide level, which become submerged twice daily for several hours. Submergence for long periods increases the likelihood that fauna colonizing the abandoned burrows may drown or asphyxiate. Hence, many faunal groups are naturally barred from recolonizing burrows abandoned by the beetle, thus fewer fungi species are expected to be inoculated in the burrows. This illustrates that both tidal amplitude and height above datum are important factors in the recolonization and survival of both faunal species and possibly the fungi in the mangrove environment.

**Fig 5:**
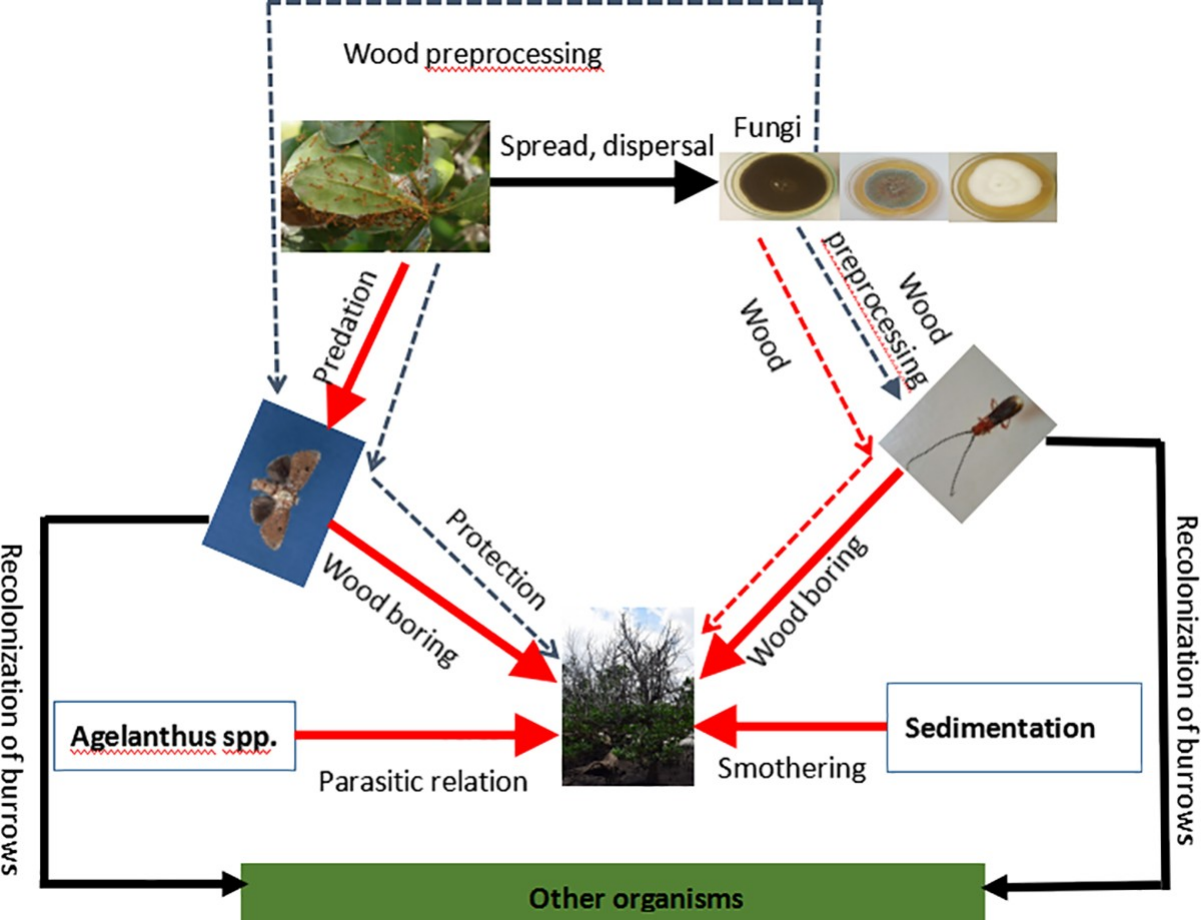
A scheme indicating the major challenges faced by *S*. *alba* and the interactions among the different organisms influencing its survival. The kind of interaction between the organisms is written next to the arrows. Blue dotted lines indicate an assumed beneficial relationship between organisms, a black line indicates a relationship thought to be beneficial, red lines indicate a harmful relationship, whereas a red dotted line indicates a relationship thought to be harmful.

Having only three woodborer-associated fungal species shared between the moth and the beetle in this identification may indicate a degree of woodborer specificity in choosing fungal species due to their different roles in the association. However, in this study, the relationships between these fungal species and woodborers were not yet established (Fig 5). Despite some of the fungal species having been recorded to have the ability to degrade cellulose and lignin [12,25,43,55,56], all the isolates that were tested for ligninolytic and cellulolytic activity in this study produced negative results with the methods used. This could mean that the woodborers do not rely on these fungal species to degrade the infested branches. We suggest a passive relationship where the woodborers simply assist the fungi by transporting them from one host to another, without benefit from the fungi (Fig 5). Alternatively, most of the fungi recorded in this study could have been introduced by the fauna that recolonized the burrows after the woodborers had left. If this is the case, then the fungi recorded here present more of an inventory of the fungal flora in the sampling sites than associates of the woodborers. We may conclude that fungal diversity in Mida Creek is low compared to that of Gazi Bay and the dominant fungal genus in Mida Creek is Penicillium.

It is interesting that the genus Penicillium is represented by only one species in Gazi Bay out of the 13 occurring species, whereas in Mida Creek this genus is represented by three of the eight different recorded species. The genus association with the beetle and its occurrence in Mida Creek could provide a new frontier for bioprospecting since fungal isolation from mangrove habitats had shown stronger antibacterial activity than from other marine habitats [25]. Due to this association, it would be important to first understand the benefit the beetle derives from interacting mainly with Penicillium spp. since during laboratory rearing of beetle larvae, Jenoh *et al*., [24] discovered that the beetles could not survive when fresh food (new branches of *S*. *alba*) was introduced and they speculated that the beetle relied heavily on fungi.

The fungal species documented in this study appeared predominantly in infested dead (ID) branches compared to infested recovered (IR) branches (Table 3) at 13 and 6 species, respectively. It could be that the ID branches offered more conducive habitats for colonization by different faunal groups, which in turn inoculated the burrows with a larger number of fungal species. On the other hand, recovering branches might not offer a good habitat for either new inoculation and/or the establishment of fungi that were already present. This may be due to three factors: (1) an infestation which occurred but was not fully established, (2) deposited insect larvae or pupae that were consumed by ants or other predators before they could establish or (3) the plant defence system was able to deter further infestation by the woodborer, especially the moth. A vigorous branch may not offer a good habitat for burrow recolonization by many faunal groups since in many cases where branch recovery happens, the burrows are generally short, few per infestation point and numerous ant individuals are usually present to defend the branch, which deter faunal species from colonizing IR branches and the co-inoculation of many species of fungi.

The negative results for both cellulolytic and ligninolytic activity screening could be because the fungi do not assist in pre-degrading wood for the insects but rather constitute a food item. It could also be that the woodborers have a microbial flora within their gut that support degradation of the wood tissue that they consume. If this is true, then it is highly likely that the recorded fungi observed are mainly reflecting the diverse fungal flora in the study sites rather than that the fungi are specifically associated with the woodborers. To ascertain if the latter is the case, investigation should focus on branches with active infestation. This would eliminate the possibility of studying fungi that were inoculated by fauna recolonizing the abandoned burrows.

## Conclusion

Understanding the infestation mechanisms of the insect woodborers is an essential first step towards determining meaningful management options and priorities. *Bottegia rubra* must be given more attention since once it infests a branch it kills it. *Bottegia rubra* also attacks young trees and thus it is a threat to reforestation efforts where plantations have been established. Unlike the moth, the population growth of which could possibly be controlled by one or two ant species, *B*. *rubra* has no known natural enemy with the same effectiveness as for the moth in this mangrove stand. However, the long generation time and the time taken to kill a single branch buffer its destructive effect. It appears that controlling the spread and destruction of the metarbelid moth is difficult due to the infestation mechanisms it employs. On the other hand, since *B*. *rubra* infests the topmost part of a branch moving downward, and because the infestation is almost immediately visible, removal of the larvae through active cutting of newly infested branches has proven to be a cheap, robust and effective management option. It is environmentally friendly and can be conducted with little technical know-how. Thus, if need arises, local communities can take the lead in conserving the mangrove forest adjacent to their villages and the coastline, using this method which does not destroy the entire infested branch. Our results further showed that the infestation mechanism used by the respective woodborer directly affected the fauna that recolonized abandoned woodborer galleries, possibly indirectly determining the diversity of fungal flora inoculated. Our results cannot unambiguously ascribe associations between the woodborers and identified fungi. Fungi could also have been introduced or inoculated by other fauna into the abandoned woodborer galleries. We suggest that follow-up studies should target branches with active infestation. Since both insects prevent other organisms from accessing the burrows until they abandon the branch, targeting branches with live woodborers will guarantee that any fungi found in the burrows were most likely contributed to by the woodborers, with a likely association with the insect. We also recommend further studies to investigate the presence or not and nature of microorganisms in the gut of the woodborers.

Through this work, we have outlined the mechanisms used by the two woodborers to infest *S*. *alba*, and the presence of fungi in the woodborer-infested *S*. *alba* branches has been proven and their possible role discussed.

## Supporting information

S1 File Ants occurance data. (XLSX)

S2 File Multi-toff data. (DOCX)

S3 File DNA sequencing data. (ZIP)
