# Supplementary material for: Infestation mechanisms of two woodborer species in the mangrove Sonneratia alba J. Smith in Kenya and co-occurring endophytic fungi
Source: PLoS One. 2019 Oct 4;14(10):e0221285. doi: 10.1371/journal.pone.0221285 (PMC6777984; doi:10.1371/journal.pone.0221285)
Supplement: S2 File — (DOCX) [file pone.0221285.s002.docx]

**From:** Hendrickx, Marijke [<mailto:Marijke.Hendrickx@wiv-isp.be>] 
**Sent:** maandag 30 januari 2017 16:26
**To:** Nico Koedam
**Subject:** RE: stammen / isolaten

Hallo Niko,

Hierbij de resultaten van de MALDI-TOF analyses:

-          MIDA5 REP3/1: Penicillium sclerotiorum

-          GAZI REP3: Not in database

-          PH 3/1: Talaromyces diversus

-          PH: Aspergillus tubingensis

-          MIDA5 REP1: Aspergillus tubingensis

-          MIDA5 REP3b: Not in database

-          PH3: Aspergillus tubingensis

-          PH2: Not in database

-          KAIRO 3: Talaromyces australiis

-          KAIRO 2: Aspergillus japonicus

-          JUDY 1: P. sclerotiorum

-          MIDA5 Rep3a: Not in database

-          MIDA3 rep3: P . citrinum

Kan je me laten weten wat we nu verder met de stammen moeten doen?

Voor opname in de collectie zal ik dus wat meer info nodig hebben.

Groetjes

Marijke

| Marijke Hendrickx, PhD  Head of scientific service  Mycology & Aerobiology  Wetenschappelijk Instituut Volksgezondheid Institut Scientifique de Santé Publique  Scientific Institute of Public Health [www.wiv-isp.be](http://www.wiv-isp.be/) |  |
| --- | --- |
